# Possible Estrogen Dependency in the Pathogenesis of Branchial Cleft Cysts

**DOI:** 10.1155/2017/1807056

**Published:** 2017-12-19

**Authors:** Jan D. Raguse, Ioannis Anagnostopoulos, Christian Doll, Max Heiland, Korinna Jöhrens

**Affiliations:** ^1^Department of Oral and Maxillofacial Surgery, Charité – Universitätsmedizin Berlin, Corporate Member of Freie Universität Berlin, Humboldt-Universität zu Berlin, and Berlin Institute of Health, Berlin, Germany; ^2^Institute of Pathology, Charité – Universitätsmedizin Berlin, Corporate Member of Freie Universität Berlin, Humboldt-Universität zu Berlin, and Berlin Institute of Health, Berlin, Germany

## Abstract

**Background:**

Even though branchial cleft cysts are currently accepted as a congenital anomaly, there is often a long delay until clinical presentation; branchial cleft cysts classically appear in the second to fourth decade of life. Our observation of their occurrence in three pregnant women encouraged us to contemplate a possible hormonal influence.

**Methods:**

Immunohistological analysis was performed for the evaluation of the estrogen receptor alpha (ER*α*) in paraffin-embedded tissue specimens of 16 patients with a diagnosis of branchial cleft cyst, with three of them being pregnant.

**Results:**

Expression of ER*α* was detected within epithelial cells only in branchial cleft cysts in pregnant females; moreover, higher growth fractions (Ki-67/Mib1) were found.

**Conclusion:**

The fact that the estrogen receptor was expressed only in pregnant women, in contrast to 13 investigated cases, may suggest that the high level of estrogen in pregnancy is a possible explanation for the spontaneous growth of branchial cleft cysts.

## 1. Background

A broad spectrum of pathologies manifest themselves by swelling in the neck as an early symptom. Beyond frequent reasons such as abscesses or neoplasms, a branchial anomaly is causative in only 0.2% of all cases, with an overall occurrence rate of 1 : 3500–4000 [1]. Of these, second branchial cleft cysts are the most common, seen in 90–95% of cases, classically presenting as a solitary, painless mass in the neck near the anterior border of the sternocleidomastoid muscle [2]. According to the current literature, females and males are equally affected. Although different theories on the origin of branchial cleft cysts were discussed for decades, today it is widely accepted that this anomaly stems from congenital remnants of the branchial arch [3, 4]. Although it is a congenital lesion, the first manifestation typically occurs between the second and third decades of life [5]. On the other hand, in a precise anamnesis, patients often report a history of intermittent swelling and tenderness, particularly associated with a banal infection like a cold. However, as far as we know, there is no actual report on the specific factors that may trigger cyst growth, and particularly no reports were found about increased incidence during pregnancy.

Our clinical observation of the occurrence of branchial cleft cysts in three pregnant women, presenting in our department within two years, gave rise to the question of whether estrogen, which is elevated during pregnancy, is able to induce the growth of these branchial cleft cysts. Therefore, we performed an immunohistological study analyzing the growth fraction (Ki67/Mib-1) and the estrogen receptor alpha (ER*α*) expression pattern in these three cases and compared them to branchial cleft cysts resected from nonpregnant women and men of different ages.

## 2. Material and Methods

### 2.1. Ethics Statement

The Ethics Committee of the Faculty of Medicine Charité, Medical University Berlin, approved this study (EA2/038/15).

### 2.2. Patients

**Figure 1:**
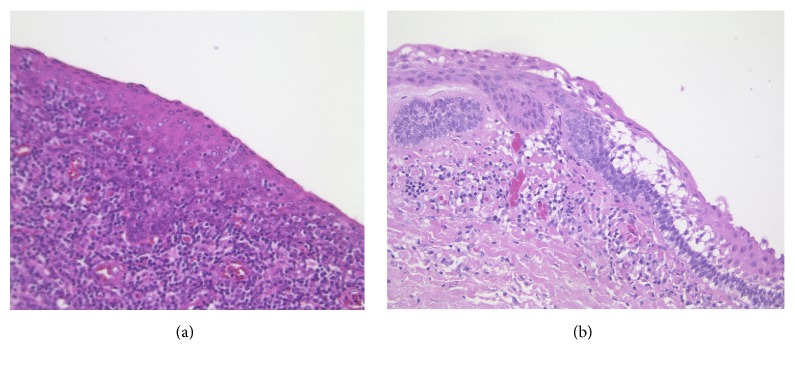
H&E staining (20x) of a branchial cleft cyst of a pregnant woman (a) and a 48-year-old male patient (b).

For the purpose of this study, we investigated paraffin-embedded tissue specimens from 16 patients with a diagnosis of branchial cleft cysts. Seven patients were female, with three of them being pregnant (aged 25, 26, and 28) along with three nonpregnant (aged 20, 28, and 51) women; the remaining female subject was a 4-month-old child. The other nine patients were males of different ages (ranging from 8 to 82 years). Histological analysis confirmed the diagnosis of a branchial cleft cyst in all of these cases (Figures 1(a) and 1(b)).

**Figure 2:**
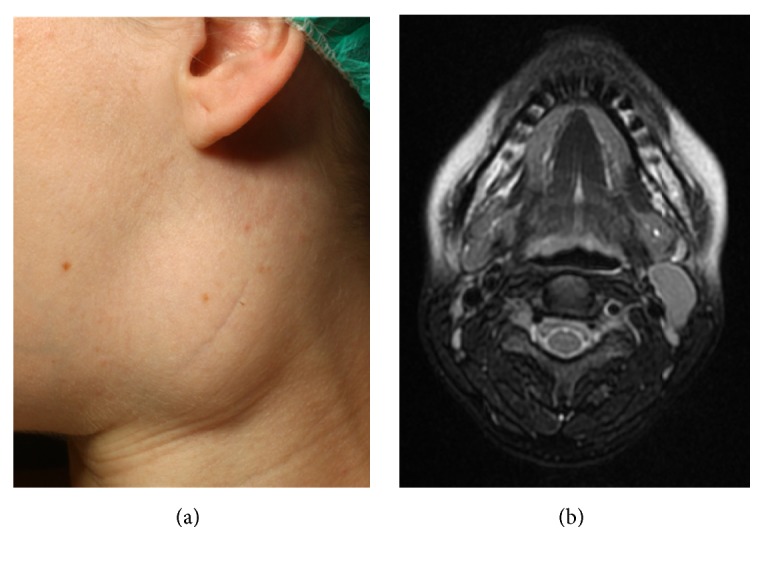
Clinical picture of a pregnant patient with a branchial cleft cyst on the left side of the neck (a) and the corresponding MRI showing the cystic mass in the vessel/nerve sheath (b).

Within a period of 24 months, the three pregnant women presented consecutively with a growing, firm elastic mass in the anterior triangle of the neck (Figure 2(a)). By MRI, cystic lesions of 32 × 28, 60 × 30 and 63 × 60 mm were seen (Figure 2(b)).

**Figure 3:**
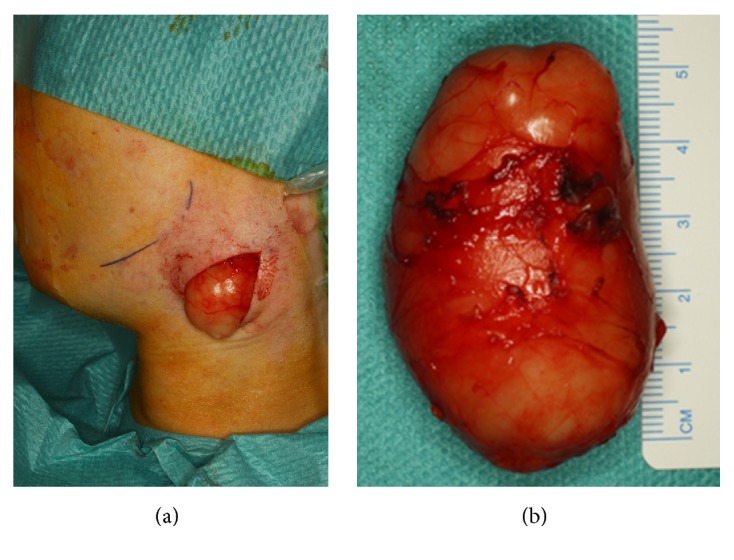
Clinical picture after evolution of the branchial cyst (a) and clinical picture showing the complete specimen (b).

In all cases, the cysts were extirpated under general anesthesia without any complications (Figures 3(a) and 3(b)). The lymphoid tissue in the cyst stroma was organoid in two cases, while in the third it was partially destroyed by a severe inflammation. The diagnosis of lymphoma could be excluded by conventional as well as immunohistochemical staining.

### 2.3. Immunohistochemical Analysis

Immunohistochemical analysis was performed on 4-mm sections obtained from formalin-fixed paraffin-embedded material. The primary antibodies used for the evaluation were antiestrogen receptor alpha (clone SP1) from Ventana Medical Systems (Tucson, USA) provided as a ready-to-use antibody and Ki-67 clone Mib-1 obtained from Dako (Glostrup, Denmark) applied at a concentration of 1 : 100. The sections were dewaxed and subjected to an antigen retrieval protocol within a BenchMark Ultra instrument (Ventana Medical Systems) followed by incubation with primary antibodies. Bound antibodies were visualized using the streptavidin–biotin–peroxidase method and diaminobenzidine as the chromogen (ultraView Kit, Ventana Medical System). For positive controls, tissue microarray sections from breast carcinoma cases were used. As a negative control we incubated tissue sections with commercially obtained IgG-antibodies of the same subclass as the monoclonal antibodies used for Ki-67/MiB1 and ERalpha.

## 3. Results

### 3.1. Branchial Cleft Cysts Arising during Pregnancy

**Figure 4:**
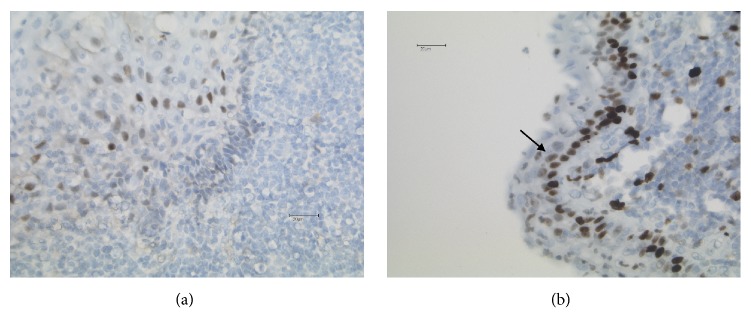
Immunohistological staining (20x). (a) Nuclear expression of estrogen receptor alpha in the basal and middle layer of the squamous epithelium lining a branchial cleft cyst arising in a pregnant female patient. (b) Strong nuclear expression of Ki-67 (40x) in the entire basal layer as well as focal expression in the middle layer of the epithelium (arrow).

In one of the three affected pregnant women, the epithelium could not be evaluated as it was totally destroyed by inflammation. The other two cases weakly expressed ER*α* in nuclei of a portion of the epithelium (Figure 4(a)). Ki-67/Mib1 positive cells were present not only in all cells of the basal layer but also focally in the middle layer of the cyst epithelia (Figure 4(b)). Whereas the epithelial cells in the branchial cysts of pregnant women exhibited a proliferation index of 21 to 30%, this index ranged in the cysts of nonpregnant woman and men from 3 up to 9%.

### 3.2. Branchial Cleft Cysts in Nonpregnant Female Patients and in Males

**Figure 5:**
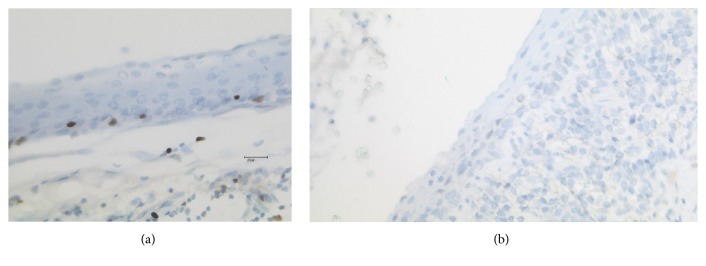
Immunohistological staining. (a) Few cells express Ki-67 (40x) in the epithelium of the branchial cleft cyst in a male patient. (b) Complete absence of estrogen receptor alpha expression in the epithelium in the same case (20x).

The growth fraction was lower than that in the two cases of pregnant women: 7/13 cases exhibited just a few Ki-67/Mib-1 positive cells in the basal layer (Figure 5(a)). In four further cases, most of the cells in the basal layer expressed Ki67/Mib-1, and in the remaining two cases (one man and one woman) a discontinuous expression pattern in the basal layer and in some cells of the middle layer was observed. All 13 investigated cases of this group of patients showed no expression of ER*α* in the epithelia lining the cysts (Figure 5(b)).

## 4. Discussion

This study was performed to provide an answer to the hypothesis that estrogen is able to induce epithelial proliferation in branchial cleft cysts, thus leading to their enlargement during pregnancy as observed in three consecutive cases. This theory was supported by the report of a patient with an enlargement of a branchial cleft cyst after child-birth followed by spontaneous resolution without intervention, perhaps as a result of the hormonal changes that occur after pregnancy [6].

Both evaluable cases of pregnant women showed weak nuclear expression of the ER*α* in the epithelial lining of the cysts, whereas all other investigated cases of branchial cleft cysts arising in males or nonpregnant females did not show any expression of this receptor. Moreover, most of the cysts arising in males or nonpregnant females (with the exception of two cases) showed a lower proliferative activity than those detected during pregnancy.

It is well known that estrogen plays an important role in cell proliferation and growth within and outside the reproductive system [7]. These effects are mediated by the ER*α* and estrogen receptor beta (ER*β*), which act as hormone-inducible transcription factors [8] and belong to the class 1 nuclear receptor superfamily [9]. During pregnancy, the estrogen 17*β*-estradiol circulates in the blood; thereby, this free steroid diffuses in and out of all cells [10]. When estrogen binds at ER*α* or ER*β* at the cell surface, the receptors dimerize and bind with high affinity in the regulatory regions of their target genes [11, 12].

Many studies regarding the regulatory effects of estrogen in different organs outside the reproductive system, especially in the liver, brain, and retinal endothelial cells [8, 13, 14], have been published. Furthermore, it has been recognized that estradiol-17*β* (E2) controls epithelial proliferation via cross-talk between epithelial-stromal cell layers [15], although the molecular mechanism by which the stroma mediates this communication remains poorly understood [16]. In particular, the negative effects of estrogen deficiency on bone healing and their improvement by 17*β*-estradiol have been described [17]. Estrogen obviously has a regulatory effect on liver regeneration after partial hepatectomy [14, 18]. Spencer et al. identified in pregnant sheep a correlation between the loss of progesterone receptor (PR) and an increase in ER expression in the endometrial luminal and superficial glandular epithelia [19]. Moreover, Oboti et al. demonstrated that pregnancy and estrogen enhance the proliferation of neural progenitor cells in the vomeronasal sensory epithelium. Both of these studies led to the hypothesis that pregnancy induces a higher level of ER in epithelial cells and that estrogen is able to influence cell proliferation in a positive way [20]. These data are in line with our observations.

As described, estrogen can induce the expression of ER*α* in various cell types such as liver cells [14] and breast cancer cells [21]. Based on these findings, it might be possible that high levels of estrogen during pregnancy may lead to an (unspecific) increase in ER*α*-expression within epithelial cells in branchial cleft cysts. Consequently, the observations presented in this study might be a nonspecific phenomenon in pregnant women. However, the fact that in 3 out of 7 female patients the branchial cleft cyst became clinical apparent during pregnancy supports the hypothesis that estrogen might have influenced the growth of the cysts through ER*α*. The exact reason for the different receptor expression between pregnant and nonpregnant patients cannot be concluded from this study. The evaluation of more patients might lead to further information.

The effect of pregnancy on tumors remains a major concern today. In the current literature, there are a few reports mentioning an association between steroid hormones/ER and soft tissue tumors (liposarcoma, hidradenoma) [22, 23]. These findings also support our hypothesis in general.

## 5. Conclusions

Although we could demonstrate ER*α* expression in the epithelial lining of branchial cleft cysts in only two cases arising in pregnant females, in contrast to all other cysts arising in males and nonpregnant females, this is nevertheless the first study to postulate a tissue-based explanation for the up to now unexplained growth of branchial cleft cysts in pregnant women.
